# Combining Optical Control and Geometrical Optimization for Efficient Control of Competing Molecular Photoinduced Processes Far from the Ground State

**DOI:** 10.1021/acs.jctc.5c00609

**Published:** 2025-06-20

**Authors:** David Veintemillas, Bo Y. Chang, Ignacio R. Sola

**Affiliations:** † Departamento de Quimica Fisica, 16734 Universidad Complutense, Madrid 28040, Spain; ‡ School of Chemistry, Seoul National University, Seoul 08826, Republic of Korea

## Abstract

The yield of a photochemical process can be maximized by optimizing the driving fields, such as in optical control, or the initial wave function, as in geometrical optimization. We combine both algorithms in an iterative process, showing very fast convergence and great improvement in the yields, as applied to driving population to the second excited state of the molecular hydrogen cation through the first excited dissociative state by a pump–pump scheme. The results reveal the impact of the initial vibrational coherences in photoinduced processes that occur at nuclear configurations very far from the ground state, or that are even mediated by processes in the continuum. On the other hand, depending on whether we maximize the total electronic population (that mainly dissociates) or the bound population, the initial wave functions change considerably, involving nodal patterns in the position or in the momentum representations, respectively, that lead to different dynamics.

## Graphical abstract

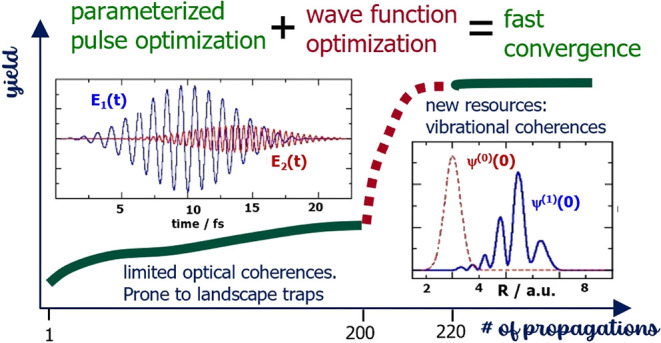

## Introduction

The development of quantum control algorithms^1–10^ and pulse-shaping techniques,^4,11,12^ has led to many theoretical and experimental studies controlling the photofragmentation of complex molecules driven by cleverly designed lasers.^11–19^ Photoinduced processes depend on the characteristics of the excitation, but also on the initial conditions. While extended efforts have been devoted to the first question, quite fewer studies have focused on the latter, where the role of the initial conditions was typically limited to evaluate the impact of starting from different vibrational states.

However, already in 1986, Brumer and Shapiro proposed to start from an initial superposition state to control photodissociation reactions by constructive and destructive interference.^20^ More recently, the formalism to control reactive scattering was further developed.^21,22^ Although in principle, the process of preparing such starting superposition state, followed by its excitation, could all be included in the dynamical optimization, it is reasonable to separate the two events, as they involve different degrees of freedom (vibrational vs electronic motions) and as such, typically require different laser frequencies. One can conceive of different control protocols for the preparation stage of the reactants, versus the scattering or photoinduced process, as substantiated in several quantum control proposals where a short and strong infrared (IR) pulse ignites the dynamics in the ground potential, before the optical or ultraviolet (UV–vis) pulse moves the wave packet to the excited electronic state where the reaction or photophysical process occurs.^23–28^ Alternatively, the original Tannor–Rice scheme proposed using a UV–vis pulse to generate the dynamics in the excited electronic state, followed by a dump pulse (also in the UV–vis) retrieving the wave packet in the ground potential, where the reaction of interest unfolded.^29^

While the IR followed by the UV–vis pulse is a particularly simple realization of a scheme to prepare the initial state, where the IR pulse creates momentum along the permanent dipole of the molecule, more elaborate procedures could in general be necessary, using for instance modulated IR pulses, or controlled Raman processes. Liberating the preparation of the initial state from a particular choice of laser protocol, enables the formulation of the optimization of the initial wave function as a variational problem. This is the basis of the so-called geometrical optimization (GO) approach, which has been recently applied to different processes, including maximizing the yield of absorption^30–32^ and photodissociation, controlling its total yield^33^ as well as the branching ratio^34^ to control isomerization reactions^35^ or to accelerate adiabatic passage.^36^ In most single-photon processes, great improvement in the yields could be observed, which allowed for simple interpretations of the control mechanism.^33^ In this work we will use a combination of laser optimization and initial wave function optimization to control a two-photon process that occurs at geometries and energies very far from the initial state. Testing the applicability of the geometrical optimization in these conditions will strengthen the importance of the initial vibrational coherences in guiding the dynamics.

Our goal will be to induce selective photodissociation in an electronic channel or, more importantly, the preparation of a vibrational wave packet in a highly excited metastable electronic state, where vibrational population remains trapped embedded in a continuum. The states that we are interested in involve large bond distances and large polarizabilities, which can be used as precursors to indirectly control reactive scattering events or a doorway to manipulate electron dynamics.^37–47^ In particular, we will focus on reaching the second excited electronic state of the hydrogen molecular cation, B ^2^Σ_*g*_. There is a vast literature referring to the correlated effects of electron and nuclear motion during dissociation in the first electronic state of H_2_^+^, A ^2^Σ_*u*_, mainly concentrating on the effects on electron localization^48–54^ but also exploring wave packet stabilization through bond hardening.^55–57^ One disadvantage of the control of nuclear wave packets in this electronic state is that the prepared wave packet can only remain trapped as long as the pulse is switched on. Going to the next excited state provides the support of vibrational levels, which do not decay within the lifetime of the external field, but adds the difficulty of stopping the ladder of excitation up to ionization.

The first step in our optimization protocol is to optimize the pulses. To do so, we parametrize both pump pulses and either use a simple line-search approach to find the pulse amplitudes and the time-delay that maximize the yields, or we use a gradient-based quantum-optimal control algorithm to find the optimal pulse parameters. The main goal of our work is to find out whether the geometrical optimization performed after the optical optimization can still improve the desired yields. From the point of view of coherent control, this reveals the crucial influence of the initial vibrational coherences in driving the dynamics, which show that even when the field is optimal for the given initial state, the initial state is not optimal for the field. There is still room for improvement by facilitating the dynamics in the ground potential.

In addition, the analysis of the optimized initial wave functions in the Wigner representation hints at different mechanisms responsible for the control of the dynamics, which are encoded in the nodal patterns either in the position or in the momentum representation, serving in our case to outperform ladder excitation or to avoid dissociation.

From the methodological perspective, our results show that geometrical optimization is a simple and fast procedure which can be used to quickly improve the results of the dynamical optimization, perhaps avoiding the usual local maxima or traps in optimal control theory, which are prevalent when the pulses are parametrized. Finally, by combining GO with dynamical optimization in an iterative procedure, we show a very fast convergence toward the maximized yields.

## Theory

### Model Hamiltonian

As an illustrative model where we can test the performance of the geometrical optimization under difficult conditions, we simulate a double-pump excitation to the *V*_3_ electronic state of H_2_^+^ using a Hamiltonian consisting of the ground and first five excited electronic states (|*j*⟩, *j* = 1,···,6), where the potential energy matrix operator is given by

1 $$\mathrm{V̂}=\overset{6}{\underset{i=1}{\sum }}V_{i}\left(R\right)\left|i\right\rangle \left\langle i\right|+\overset{6}{\underset{i=1}{\sum }}\overset{6}{\underset{j\neq i}{\sum }}\mu_{ij}\left(R\right)\left[\varepsilon_{1}\left(t\right)+\varepsilon_{2}\left(t\right)\right]\left|i\right\rangle \left\langle j\right|$$

For later reference, we will call Ψ­(*t*) (with capital letter) to the full nuclear wave function with the 6 components (the wave packets in each electronic state), and **μ** (with bold-type letters) to the dipole moment matrix. Each field is parametrized as ε_*k*_(*t*) = *E*_*k*_ *f*[(*t* – *t*_*k*_)/σ_*k*_]­cos­[ω_*k*_(*t* – *t_k_*)] (*k* = 1,2), with *f*(*t*′) = 0.42 – 0.5 cos­(2π*t*′) + 0.08 cos­(4π*t*′) in the domain 0 ≤ *t*′ = (*t* – *t_k_*)/σ_*k*_ ≤ 1. The shape is chosen to approximately reproduce a Gaussian function supported on a finite time interval. Notice that the field is the sum of the two pump pulses as we do not apply the rotating-wave approximation.^58,59^

The potential energy curves *V*_*j*_(*R*) and the most important dipoles μ_*jk*_(*R*) are shown in Figure 1. The potentials are clustered in pairs that dissociate to the same atomic states (1s for *V*_1_(*R*) and *V*_2_(*R*), 2p_*z*_ for *V*_3_(*R*) and *V*_4_(*R*), and 2s for *V*_5_(*R*) and *V*_6_(*R*)). They form charge resonances, so the dipoles μ_12_(*R*), μ_34_(*R*) and μ_56_(*R*) increase linearly for large bond distances. All other transition dipoles (not shown) are also included in the calculations. The larger dipoles involve one-stepladder excitations *V*_*j*_ → *V*_*j*+1_ with (smaller) transient dipole μ_23_(*R*) corresponding to the 1s → 2p_*z*_ atomic dipole at large distance, and μ_45_(*R*) correlating to the 2p_*z*_ → 2s atomic excitation at large *R*. The strong dipoles make harder to control the dynamics (and avoid further excitation) at large internuclear distances, where the ionization probability also increases. To estimate the ionization probability, we performed dynamical calculations using a 2*D* time-dependent Schrödinger equation (TDSE) for an electron aligned with the nuclei, interecting via a soft-core Coulomb potential^60,61^ observing in most cases negligible ionization probabilities, smaller than 0.1%.

**Figure 1:**
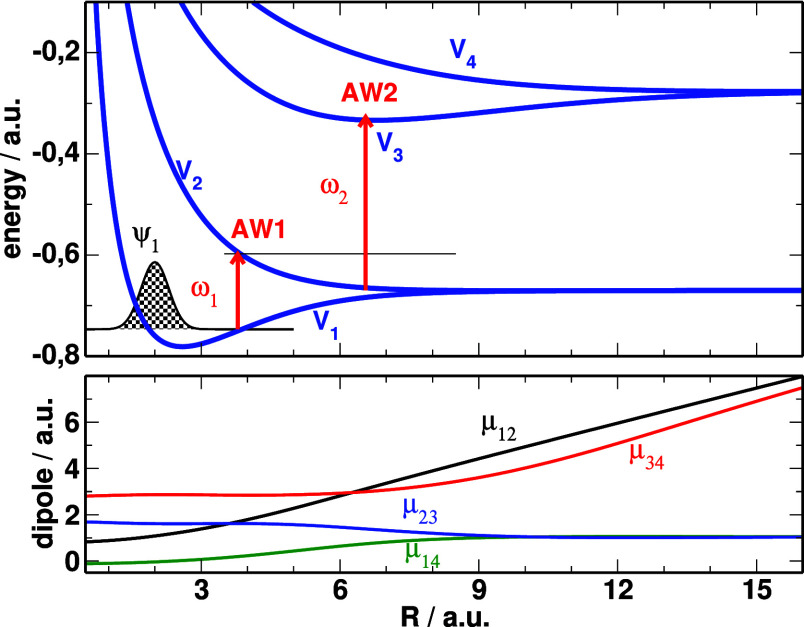
Potential energy curves (top) and dipoles (bottom) of H_2_^+^ included in the calculations. We mark the absorption windows AW1 and AW2, where most of the wave packet transfer between *V*_1_ → *V*_2_ and *V*_2_ → *V*_3_ take place, when the dynamics starts from a Gaussian centered at *R* = 2.0 au, ψ_1_, representing the state after sudden ionization of the parent molecule.

Although the following optimizations were performed in the reduced model with 6 electronic states, for consistency, the states and dipoles were calculated using the Fourier-grid Hamiltonian^62^ (FGH) method applied to the soft-core Coulomb Hamiltonian. The vibrational wave functions for the bound *V*_1_(*R*) (ϕ_*j*_(*R*)) and *V*_3_(*R*) (φ_*j*_(*R*)) electronic states were also computed using the FGH for a grid of 768 points from *R* = 0.5 to 38.85 au in the bond coordinate. Then we solved the TDSE for the coupled 6-electronic potentials in the grid, using the split-operator method.^63,64^ Finally, the dynamics with optimized pulses and initial wave functions were solved again in the 2*D* model, showing very small deviations with the reduced model results, except for a few examples indicated in the paper.

### Optimization Procedure

We aim to maximize the yield of a quantum process, computed as the expectation value of a projector operator *P̂*. In this work, this operator can be the total electronic population in *V*_3_ at final time, *P̂*_1_ = |3⟩⟨3|, or its bound population. In the latter case the projection operator is a sum over all (*N*_2_) bound vibrational eigenstates of *V*_3_, ${\mathrm{P̂}}_{2}=\overset{N_{2}}{\underset{j}{\sum }}\left|3⊗\varphi_{j}\right\rangle \left\langle \varphi_{j}⊗3\right|$ .

We can define the yields as the expectation values of the respective projection operators at time *T*. In the Heisenberg picture, $\chi_{j}=\left\langle \Psi \left(0\right)\right|{\mathrm{P̂}}_{j}^{H}\left(T\right)\left|\Psi \left(0\right)\right\rangle$ where

2 $${\mathrm{P̂}}_{j}^{H}\left(T;\varepsilon_{1}\left(t\right)+\varepsilon_{2}\left(t\right)\right)={Û}^{-1}\left(T\right){\mathrm{P̂}}_{j}Û\left(T\right)$$

with *Û* the time evolution operator, which depends on the pulses ε_1_(*t*) + ε_2_(*t*). The yield also depends on the initial wave function, Ψ(0) ≡ (ψ, 0, 0, 0, 0, 0)^T^, with initial components only on the *V*_1_ potential, ψ_1_(*R*). Therefore, χ_*j*_ becomes an explicit functional of Ψ(0) and an implicit functional on the fields ε_*k*_(*t*).

Finding the maxima with respect to variations in ψ­(*R*), ∂χ_*j*_/∂ψ = 0 leads to the geometrical optimization equations. Limiting the form of ψ as a superposition of *N*_*b*_ vibrational eigenfunctions of *V*_1_, $\psi =\overset{N_{b}}{\underset{v}{\sum }}c_{v}\phi_{v}$ conditioned upon normalization, gives the eigenvector equation for the optimal coefficients,^10,30,31^

3 $$\overset{N_{b}}{\underset{v}{\sum }}S_{v^{\prime }v}c_{v}=\chi \overset{N_{b}}{\underset{v}{\sum }}c_{v}$$

with

4 $$S_{v^{\prime },v}=\left\langle \Psi_{v^{\prime }}\right|{\mathrm{P̂}}^{H}\left(T\right)\left|\Psi_{v}\right\rangle$$

where Ψ_*v*_ = (ϕ_*v*_, 0, 0, 0, 0, 0)^T^, that is, the dynamics is started from the *v* eigenstate of *V*_1_. To choose the optimal initial wave function, we pick the eigenvector with largest eigenvalue, which will depend on the projection operator chosen, and the fields, ${\mathrm{P̂}}_{j}^{H}\left(T;\varepsilon_{1}\left(t\right)+\varepsilon_{2}\left(t\right)\right)$ (succinctly written as *P̂* ^*H*^(*T*) in the eq 4 above to refer to both functionals, avoiding too many indexes).

On the other hand, finding the maxima with respect to variations in the fields ε_*k*_(*t*), ∂χ_*j*_/∂ε_*k*_(*t*) = 0 leads to the quantum optimal control equations. We parametrize the fields as a function of a set of parameters ζ_*l*_ (the amplitudes and time delay between the pulses) and explore how χ_*j*_ varied as *Û*(*T*; ζ_*l*_) changed by linear changes in each parameter. The simple procedure is explained in the Results section. Alternatively, quantum optimal control algorithms will be used to find the parameters where ∂χ_*j*_/∂ζ_l_ = 0. In this case we use a gradient based approach, computing^65,66^

5 ∇ζlJ=−∑k2[2Im(⟨Ψ̃(t)|μ|Ψ(t)⟩)+αϵk(t)]∂ϵk(t)∂ζl

where Ψ̃(*t*) is the auxiliary function, defined at final time as Ψ̃(*T*) = *P̂*_*j*_Ψ­(*T*) to maximize χ_*j*_, and α is a penalty scalar that gauges the importance of the pulse fluency (i.e., the intensity of the pulses) in the functional. The evaluation of the gradient requires two propagations (those of Ψ­(*t*) and Ψ̃(*t*)). Once the gradient is known, the parameters are changed in the direction of the gradient, $\zeta_{l}^{\left(1\right)}=\zeta_{l}^{\left(0\right)}+s\nabla_{\zeta_{l}}J$ , where *s* is the length that one needs to move along the gradient, which can only be found by further propagating Ψ­(*t*) with different choices of *s* until the maximum in found. This makes the algorithm slow and typically prone to getting stuck at local maxima. More elaborated approaches based on, e.g., conjugate gradients, are also possible.^65,66^

Because in this work we combine the GO and dynamical optimization sequentially and then iterate the process, to keep track of the solution, we label the “step” in the algorithm with a superindex. We choose an initial Gaussian function ψ^(0)^(*R*) from which we first optimize the pulses, finding $\varepsilon_{k}^{\left(1\right)}\left(t\right)$ , and then improve the initial wave function ψ^(1)^(*R*) (for the given fields). Closing the loop, the next iteration will provide improved fields $\varepsilon_{k}^{\left(2\right)}\left(t\right)$ and wave functions ψ^(2)^(*R*), until convergence. We will refer to χ_1_ or χ_2_ as the goals. With a superindex, $\chi_{1}^{\left(i\right)}$ and $\chi_{2}^{\left(i\right)}$ provide the actual yields obtained in the *i*-iteration.

## Results and Discussion

We employ a simple iterative algorithm combining dynamical and geometrical optimizations for the pump–pump scheme. To optimize the parameters of the pulses we use a line-search (dynamic) approach, explained below. More advanced optimal control algorithms could be used, but they would probably obscure the nature of the combined optimization. To optimize the initial wave function, we optimize the coefficients of the superposition of vibrational eigenstates of the ground potential.

As the Franck–Condon region of the ground vibrational state promotes the wave function on the repulsive barrier of the *V*_2_(*R*) potential at high energies (in the absorption band), it requires high-frequency photons and leads to low overall yields for the pump–pump process. To avoid this problem, our simulations without optimizing the wave function, start not from *v* = 0, but from a Gaussian wave packet that emulates the state prepared after sudden ionization, that is, at the equilibrium geometry of the parent H_2_ molecule. Then this packet can be promoted to the dissociative electronic state at the attractive barrier of the *V*_1_(*R*) potential, where the absorption window overlaps the emission band, requiring lower frequency photons that create a less energetic wave packet in the dissociative state. We call this region the AW1 (see Figure 1). It is then easier to promote this wave packet in *V*_2_ to the target *V*_3_ potential where it can overlap with the minimum of the *V*_3_ potential, at what we call the absorption window 2 (AW2, see Figure 1).

### Optimizing the Pulses

We perform a physically motivated search of the optimal pulse parameters. As the carrier frequency and pulse duration of transformed-limited pulses are difficult to change in an experiment, we will fix these parameters through out all the optimization, which consists of the following procedure: First, we launch laser-free wave packet simulations in *V*_1_ using the initial Gaussian, ψ^(0)^, and in *V*_2_ at the AW1, again using the same Gaussian, but displaced to larger internuclear distances (at the AW1). Computing the expectation bond distance ⟨*R*(*t*)⟩, we find the time it takes for the initial state to reach the AW1, *t*_1_ (which is approximately half the period of oscillation in the ground state, as the initial wave packet starts from the inner wall of the potential and the this absorption window lies in the outer wall of the potential), and the time it takes from *V*_2_ to reach the equilibrium geometry of the second excited state, *t*_2_ (which mostly depends on the starting energy in the dissociative state). We fix the duration of the first pulse (fwhm) as σ_1_ = 2*t*_1_ = 20 fs and for simplicity we also choose σ_2_ = 20 fs. The time-delay between the pulses is chosen as τ = *t*_2_ – *t*_1_. The optimal parameters obtained agree perfectly with a second-order perturbation theory analysis.

To start the dynamical optimization solving the TDSE with the pulses, we need to give initial values to the remaining parameters, defining what we may call the $\epsilon_{1}^{\left(0\right)}\left(t\right)$ and $\epsilon_{2}^{\left(0\right)}\left(t\right)$ pulses. The central frequency of the first pulse, ω_1_ = 0.1486 au, is chosen as the difference between the energies at the AW1 and the average energy of the initial Gaussian, while that of the second pulse, ω_2_ = 0.3428 au, is chosen as the energy difference between the ground state of the *V*_3_ potential and the energy at the AW1. The following optimizations will be conditioned upon the choice of these frequencies, which will not be further optimized. This is in agreement with standard laboratory implementations, where the optimization of the pulse often relies on modulation, but the carrier frequency of the ultrashort (transformed-limited) pulses is not modified. The peak amplitudes of the pulses are initially chosen to avoid multiphoton processes, *E*_*k*_ = 0.001 au (*k* = 1, 2). This set of parameters give the initial input of the optimization.

Then the parameters are optimized performing a one-dimensional linear search where a single parameter is optimized (the others remain at their previous values) choosing the value of the parameter that maximizes the total population in *V*_3_ at final time. The order in which these one-dimensional searches is performed is the following: we first optimize τ, which affects the exact energy at which the absorption of the second photon takes place. Then we optimize *E*_1_ followed by *E*_2_. Obviously, there are quite more powerful optimization schemes, but for the purpose of this work, we found the previous procedure to “convergence” fast enough, especially if the linear-search are performed in the proposed order.

In Figure 2 we show the optimal pulses $\varepsilon_{k}^{\left(1\right)}\left(t\right)$ (*k* = 1,2) and the electronic populations found through this procedure. The populations follow the sequential two-step process *V*_1_ → *V*_2_ → *V*_3_, but there is no population inversion between the ground and first excited potential, as if the window of time in which the wave packet Ψ_1_(*t*,*R*) in *V*_1_ lies in the vicinity of the AW1, cannot fully exploit the transition. Some wiggles in the populations reflect nonperturbative effects especially due to the intensity of the first pulse. In addition, as soon as the population reaches *V*_3_, it immediately climbs the ladder to *V*_4_, further reducing the possible maximum yield of excitation. Because *V*_3_ and *V*_4_ form a charge resonant state, the coupling increases linearly with distance. As the wave packet evolves toward large internuclear distances in *V*_3_ (the energy minimum in *V*_3_ is already at 6.8 au) it becomes difficult to avoid the effect of the large transient dipole. We will show that changing the initial wave function can be used to heavily favor excitation to *V*_3_ over *V*_4_. Maximizing the final population in *V*_3_ we find that the average energy of the outgoing wave packet in *V*_3_ exceeds the dissociation energy, such that almost all the population dissociates. By maximizing the bound population via the laser parameters we can at most achieve χ_2_(1) = 0.0154. However, we will be able to reduce the amount of dissociation by changing again the initial wave function.

**Figure 2:**
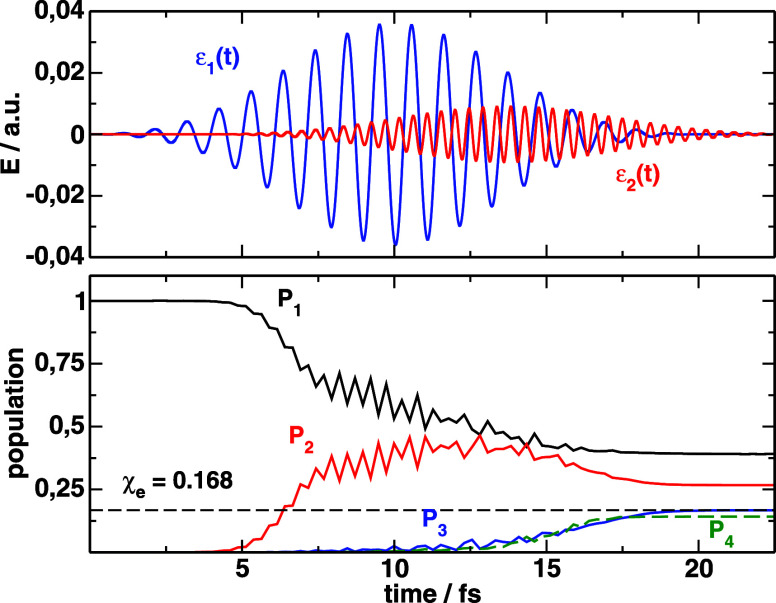
Optimal pulses that maximize electronic population in *V*_3_ after 20 fs, when the dynamics starts from a Gaussian centered at *R* = 2.0 au Below, we show the electronic population dynamics induced by the pulses.

### Optimizing the Wave Function

We now apply the geometrical optimization algorithm to find the initial wave function that maximizes the yield of population transfer to the B^2^Σ_*g*_ using the pulses $\varepsilon_{k}^{\left(1\right)}\left(t\right)$ , considering two choices: A functional that maximizes all the electronic population at final time χ_1_ and a functional that maximizes the trapped population, χ_2_. Correspondingly, we will add a subscript *j* = 1,2 to the optimized initial wave functions that maximize each yield.

Figure 3a,b shows the optimized wave function in position and quantum number representations, respectively, for $\chi_{1}^{\left(2\right)}$ , $\psi_{1}^{\left(1\right)}$ , while Figure 3c,d shows the results for $\chi_{2}^{\left(2\right)}$ , $\psi_{2}^{\left(1\right)}$ . Also shown in the figures is the initial Gaussian used to optimize the pulses in both representations. The population dynamics corresponding to the excitation from $\psi_{j}^{\left(1\right)}\left(R\right)$ (*j* = 1,2) and the pulses $\varepsilon_{k}^{\left(1\right)}\left(t\right)$ are shown in Figure 4a,b, respectively.

**Figure 3:**
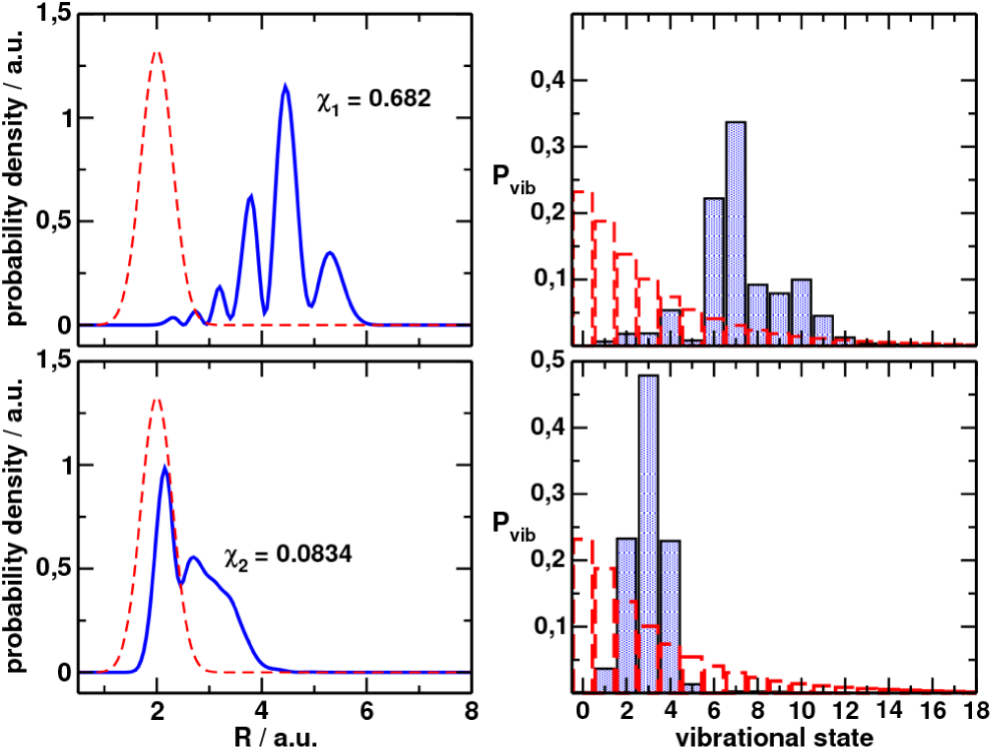
Optimized initial wave functions in the position (left) and quantum number representations (right) that maximize the final electronic population χ_1_ (top) or only the bounded population χ_2_ (bottom). In red-dashed line we show the initial Gaussian that represented the quantum state after sudden ionization of the parent molecule, ψ^(0)^, which was used to find the optimal pulses.

**Figure 4:**
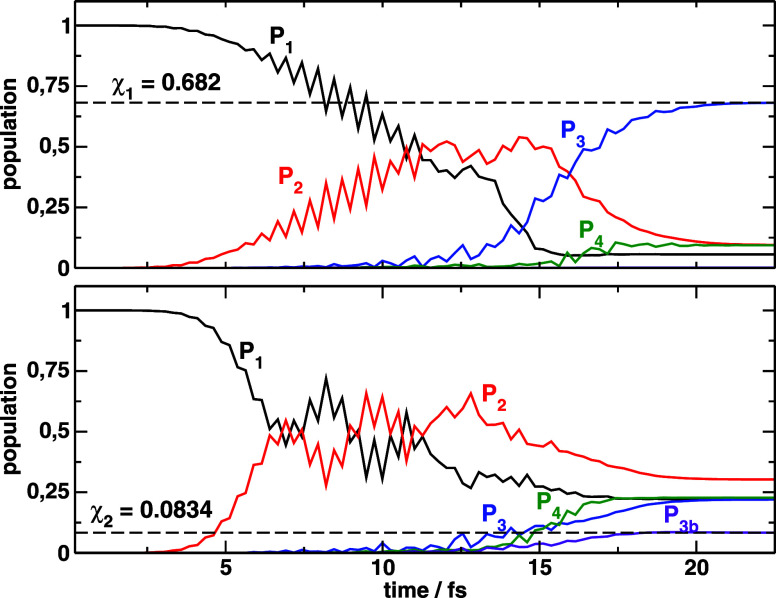
Population dynamics driven by the $\varepsilon_{k}^{\left(1\right)}$ optimal pulses starting from the wave functions $\psi_{j}^{\left(1\right)}$ , that maximize the final electronic population χ_1_ (top) or only the bounded population χ_2_ (bottom). *P*_3*b*_ refers to the vibrational (bounded) population in the *V*_3_ potential.

Regarding the optimization of χ_2_, we observe apparently similar initial dynamics, but the first pulse can excite more population to *V*_2_ starting from $\psi_{1}^{\left(1\right)}$ than ψ^(0)^, achieving population inversion. The second step of the dynamics predominantly avoids further excitation of *V*_4_. A massive improvement of the yield of excitation to *V*_3_ is achieved, with $\chi_{1}^{\left(2\right)}=0.682$ (a 4 times improvement). For this, the initial wave function has been completely modified: the vibrational populations largely favoring high vibrational quanta (around *v* = 7) with a shift in the probability distribution from the inner to the outer wall of the *V*_1_ potential, although $\psi_{1}^{\left(1\right)}$ spreads around much of the potential well. It seems that a major reason why *V*_4_ is barely populated is due to the potential energy of the initial wave function, almost 4000 cm^–1^ above that of ψ^(0)^. After $\varepsilon_{1}^{\left(1\right)}\left(t\right)$ and $\varepsilon_{2}^{\left(2\right)}$ , the wave packet in *V*_3_ has too much energy so it decouples from the *V*_3_ → *V*_4_ transition. Conversely, the shape and spread of the function maximizes the use of the transition dipole at different absorption windows throughout all the duration of the pulse (which is approximately twice the vibrational period), despite apparently not being adjusted to synchronize the peak of the pulse with the arrival of the wave packet at the AW1. We will refer again to this when we analyze the initial wave functions in phase-space. For the choice of optimization, all of the population transferred to *V*_3_ dissociates, so that the final bounded population in *V*_3_, that we call χ_2_ (or *P*_3*b*_(*T*)) although this yield is not maximized in this case, is χ_2_ = 0.

On the other hand, the dynamics obtained after maximizing χ_2_ shows several Rabi oscillations between *V*_1_ and *V*_2_ while *V*_3_ is less populated. The excitation of *V*_3_ is slightly delayed with respect to the previous case. The bounded population in *V*_3_ at final time is $\chi_{2}^{\left(2\right)}=0.0834$ (5.4 times the result obtained with ψ^(0)^) for a total of 0.22 final population in *V*_3_. Again, *V*_4_ is excited along with *V*_3_, reaching similar total populations, comparable with those achieved after ψ^(0)^. The optimized initial wave function is not as broad as $\psi_{1}^{\left(1\right)}$ , and the peak density is shifted toward smaller bond distances. In some ways $\psi_{2}^{\left(1\right)}$ is more similar to ψ^(0)^ in the position representation, but represents a packet with quite fewer contributions from vibrational eigenstates than the initial Gaussian. Basically, the wave function can be synthesized as a superposition of *v* = 2, *v* = 3 and *v* = 4 states. The position and width of the initial packet allow a faster excitation from *V*_1_ to *V*_2_ inducing Rabi oscillations. The key difference from this dynamics to the one obtained from ψ^(0)^ is that the average energy (and the potential energy) of $\psi_{2}^{\left(1\right)}$ is approximately 4000 cm^–1^ *below* that of ψ^(0)^, ensuing a final packet in *V*_3_ of similarly less energy. Thus, more population remains trapped at final time. However, the *V*_4_ state is not decoupled from *V*_3_, so that an extra photon can be absorbed. The final population in *V*_4_ is of the order of that in *V*_3_ (*P*_4_(*T*) = 0.23).

### Closing the Loop

Since the pulse was optimized for a different initial wave function, it is interesting to analyze if, once the wave function was been changed, the pulse parameters could be better adjusted to increase the desired final yields, that is, if there is still margin to improve the yields by optimizing the pulses after the geometrical optimization. This amounts to closing the loop of the geometrical (wave function) and dynamical (pulse) optimization.

Interestingly, the optimal pulses $\varepsilon_{k}^{\left(2\right)}\left(t\right)$ that maximize the yields starting from $\psi_{1}^{\left(1\right)}$ are very similar to those found previously $(\varepsilon_{k}^{\left(1\right)}\left(t\right)$ , starting from the Gaussian function) with a small 1% change in the amplitudes: an increase in the amplitude of the first pump, and a decrease in the amplitude of the second pump. Only the time delay between the pulses changes noticeably, from 3.75 to 4.88 fs. Figure 5a shows the population dynamics, which is qualitatively similar to the results shown in Figure 4a. The better adjustment of the pulse parameters ensures more population in *V*_3_ at final time (χ_1_ = 0.731) and less population in *V*_4_ (*P*_4_(*T*) = 0.076, a 20% decrease from the previous value of 0.094 population). Again, population on the bounded states remains negligible.

**Figure 5:**
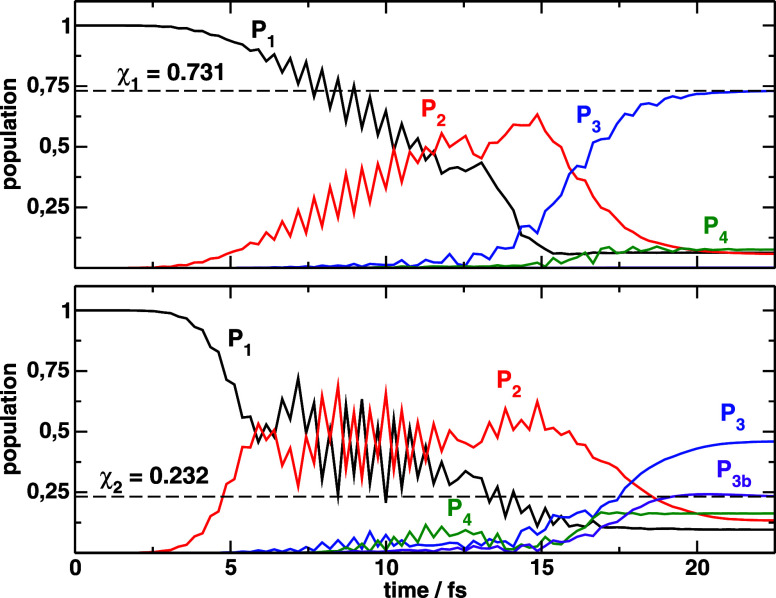
Population dynamics driven by the $\varepsilon_{k}^{\left(2\right)}$ optimal pulses starting from the wave functions $\psi_{j}^{\left(1\right)}$ , that maximize the final electronic population χ_1_ (top) or only the bounded population χ_2_ (bottom). *P*_3*b*_ refers to the vibrational (bounded) population in the *V*_3_ potential.

While we inferred that $\psi_{2}^{\left(1\right)}$ was more similar to ψ^(0)^ than $\psi_{1}^{\left(1\right)}$ , surprisingly, the optimal pulses adjusted to $\psi_{2}^{\left(1\right)}$ differ considerable from $\varepsilon_{k}^{\left(1\right)}\left(t\right)$ . The peak amplitudes of the pulses are 61% and 18% larger than previously, for the first and second pulse respectively, and the optimal time-delay is further increased to 6.25 fs. As shown in Figure 5b, the higher pulse intensities induce faster Rabi oscillations in the electronic populations, but also larger final populations in *V*_3_, with χ_1_ = 0.458 and $\chi_{1}^{\left(2\right)}=0.232$ (almost a factor of 3 larger than $\chi_{2}^{\left(1\right)}$ ). On the other hand, excitation to *V*_4_ is better discriminated than previously, with a final population of *P*_4_(*T*) = 0.16.

To better understand the mechanism behind the dynamics, we will further analyze the optimized wave functions using the Wigner representation to observe correlations between the position and momentum components of the wave function. As shown in^33^ the Wigner functions can give revealing information about the optimized wave functions. In Figure 6 we show the Wigner functions *W*_*j*_(*R*,*P*) obtained from the optimized initial wave functions $\psi_{j}^{\left(1\right)}$ , that maximize χ_*j*_, exhibiting nodal (wave-like) behavior, with negative values, which are signatures of quantum features. Interestingly, the distribution functions ρ­(*R*) and *ρ̃*(*P*) in position and momentum space, which can be obtained by integrating the Wigner function in one of the variables, are mainly broad but structureless in one of the domains, while they show oscillations in the other. In the case of *W*_2_(*R*,*P*), ρ_2_(*R*), shown in Figure 5, it is mostly a broad distribution over the interval *R* ∈ [2, 4] a.u., basically the classically allowed configuration space given the average initial energy. However, it shows nodal patterns in *ρ̃*_2_(*P*), which ultimately provoke Rabi oscillations between *V*_1_ and *V*_2_ that last several femtoseconds, delaying excitation to *V*_3_. Instead of directly dissociating, the wave packet components that reach *V*_2_ with negative momentum stay longer in the vicinity of the AW1. Since *E*_1_ is also larger, this allows for the “peculiar” Rabi oscillations through the dissociative state.

**Figure 6:**
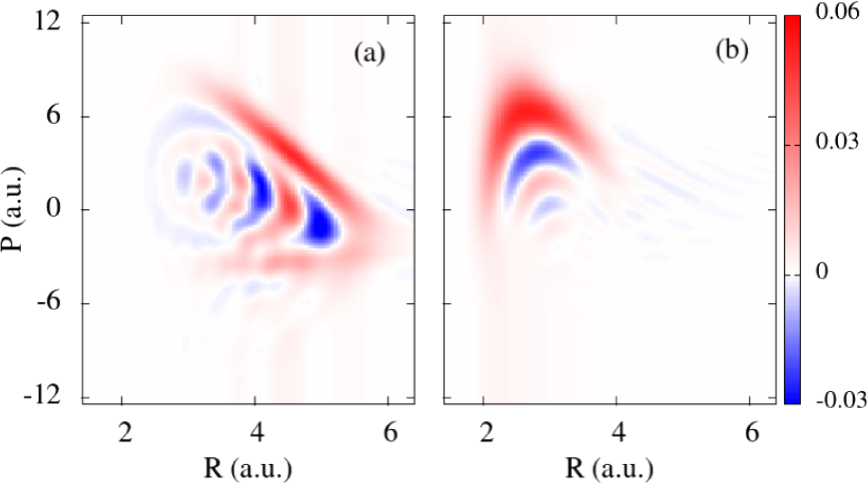
Wigner representation of the optimized initial wave functions ψ^(2)^, that maximize χ_1_ (a) and χ_2_ (b). The nodal (wave-like) patterns show quantum signatures, which are mostly present in the position representation for the $\psi_{1}^{\left(2\right)}$ function, and in the momentum representation for the $\psi_{2}^{\left(2\right)}$ function. In the first case, the maxima of the Wigner function shows some negative chirp, which implies a positive kick for the components of the function at smaller bond distances, and a negative kick for the components at larger bond distances, facilitating wave function squeezing at the AW1.

Conversely, *ρ̃*_1_(*P*) is mostly a broad Gaussian while ρ_1_(*R*) shows a nodal pattern. In addition, there is a small negative chirp in *W*_1_(*R*,*P*) that implies anticorrelation between the position and momentum components of the wave function: at shorter bond lengths positive momentum dominates, inducing a kick to larger bond distances, while at longer bond distances (for positions beyond the AW1) small negative momentum components dominate, inducing a kick to shorter bond lengths. This creates some wave packet squeezing in the position representation around the AW1 which maximizes the population transfer during a shorter time.

The iterative procedure of dynamical optimization followed by geometrical optimization can be further pursued, using now $\varepsilon_{k}^{\left(2\right)}$ as the pulses and optimizing again the initial wave functions. This second iteration gives optimal wave functions $\psi_{1}^{\left(2\right)}$ for χ_1_ and $\psi_{2}^{\left(2\right)}$ for χ_2_, which are very close to $\psi_{1}^{\left(1\right)}$ and $\psi_{2}^{\left(1\right)}$ respectively.

Figure 7a,b shows the optimized wave function in position and quantum number representations, respectively, for χ_1_, $\psi_{1}^{\left(2\right)}$ , while Figure 6c,d shows the results for χ_2_, $\psi_{2}^{\left(1\right)}$ . For reference, we also show in dotted line the distributions for the previous optimization, $\psi_{j}^{\left(1\right)}$ (*j* = 1,2). The corresponding population dynamics follows closely the results shown in Figure 5 and is not shown here. In the Supporting Information, Table S1 provides the complex coefficients of the initial wave functions expressed in the vibrational eigenstate basis.

**Figure 7:**
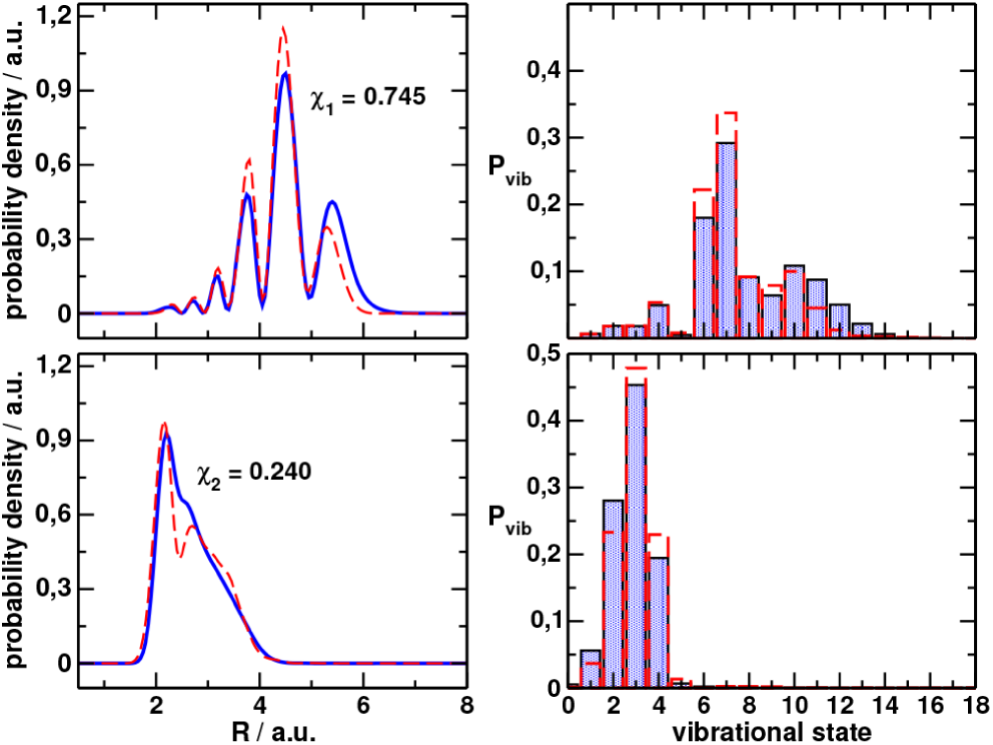
Optimized initial wave functions in the position (left) and quantum number representations (right) that maximize the final electronic population χ_1_ (top) or only the bounded population χ_2_ (bottom) obtained after the second iteration of the algorithm. In red-dashed line we show the optimal wave functions obtained after the first iteration, $\psi_{j}^{\left(1\right)}$ .

Table 1 summarizes the results of all the calculations. The optimization achieves small improvements of 1.9% in $\chi_{1}^{\left(3\right)}$ and 3.4% in $\chi_{2}^{\left(3\right)}$ over $\chi_{1}^{\left(2\right)}$ and $\chi_{2}^{\left(2\right)}$ . The iterative procedure practically saturates the optimization after two cycles, and further optimizations only improve the yield minimally (less than 1%).

**Table 1:** Total Electronic Population Transferred to *V*_3_ (*χ*_1_) and to *V*_4_ (*P*_4_(*T*)), As Well As Bounded Population in *V*_3_ (χ_2_) at Final Time, for the Different Iterations in the Combined Dynamical and Geometrical Optimization Algorithm^a^

	*P̂*_1_			*P̂*_2_		
iteration	χ_1_	χ_2_	*P*_4_(*T*)	χ_1_	χ_2_	*P*_4_(*T*)
(1) ψ^(0)^ε^(1)^	0.168	0.015	0.142			
(2) ψ^(1)^ε^(1)^	0.682	0.001	0.094	0.220	0.083	0.228
(3) ψ^(1)^ε^(2)^	0.731	0.001	0.076	0.458	0.232	0.163
(4) ψ^(2)^ε^(2)^	0.745	0.001	0.065	0.441	0.240	0.166
(1) ψ^(0)^ε^(1)^	0.419	0.004	0.080	0.355	0.193	0.101
(2) ψ^(1)^ε^(1)^	0.864	<0.001	0.064	0.571	0.550	0.098

a The first entries were obtained by optimizing 3 pulse parameters by linear search. The last entries were obtained by using a gradient-based optimal control algorithm.

While the linear-search approach can be used to avoid local traps in the landscape of the control as a function of the pulse parameters, it can only be used for a very limited number of parameters. In principle, one could expect to achieve higher yields using optimal control techniques. To avoid mixing the role of the initial vibrational coherences, explored with geometrical optimization, in the set of parameters explored by the optimal algorithms, we use a gradient-based approach where the pulses are parametrized^65,66^ as previously, but we optimize the full set of 7 pulse parameters (amplitudes, frequencies, pulse durations and the time delay between the pulses, as the first pulse is fixed to start at *t* = 0). Hence, these optical pulses will be mainly responsible for creating electronic coherences (although strong-field effects do alter the potentials).

Starting from different initial parameters (close to those in $\epsilon_{k}^{\left(0\right)}\left(t\right)$ ) and using different values for the penalty parameter α on the pulse fluency,^67^ the best result for χ_1_ after the first iteration starting from the initial Gaussian wave function, was $\chi_{1}^{\left(1\right)}=0.4754$ . (Similar results, with $\chi_{1}^{\left(1\right)}$ larger than 0.40 were found with other initial parameters.) After the geometrical optimization, the yield increased to $\chi_{1}^{\left(2\right)}=0.750$ . We found higher yields $\left(\chi_{1}^{\left(2\right)}=0.864\right)$ when $\chi_{1}^{\left(1\right)}$ was smaller $\left(\chi_{1}^{\left(1\right)}=0.419\right)$ . For χ_2_ the best result was $\chi_{2}^{\left(1\right)}=0.193$ , which increased to $\chi_{2}^{\left(2\right)}=0.550$ after the geometrical optimization. In all cases the gradient-based approach could not further improve the pulses, so convergent results were obtained at the second iteration. As previously indicated, it should be noted that gradient-based optimization typically requires solving many times the TDSE, which makes the process computationally demanding. Our best results required 34 propagations for $\chi_{1}^{\left(1\right)}$ and 377 for $\chi_{2}^{\left(1\right)}$ . On the other hand, the geometrical optimization only needs solving Ψ_*v*_(*t*) for all the elements in the basis. Including all bound vibrational states in *V*_1_ implies *N*_1_ = 24 propagations, but similar results could be obtained choosing the lowest 15 states, as the participation of the highest vibrational states in the optimized wave functions is negligible.

Figure 8 shows the population dynamics with the best results, obtained after propagating the dynamics using the full 2D Hamiltonian. The optimal yields are shown in Table 1, while the optimal pulse parameters for both the linear search approach and the gradient-based optimal control, are shown in Table 2. The complex coefficients of the initial wave functions expressed in the vibrational state basis are shown in Table S2. *P*_+_ depicts the sum of the probability of ionization and the population of the higher excited states not included in the model Hamiltonian, that was used to find the optimal pulses and optimized initial wave functions. As observed, only for the optimal pulses that maximize χ_2_, which involve high intense pulses (*E*_1_ = 0.07475 au, implying peak intensities close to 200 TW/cm^2^) the model Hamiltonian does not provide fully quantitative results. In the latter case, *P*_+_ raises to 0.40, so *P*_3*b*_(*T*) = 0.30. For comparison, we also show the bound population integrating the Schrödinger equation with the model Hamiltonian (*P*_3*b*_(*opt*)). Again, the predictions of the model are qualitatively correct, if we renormalize the result by the total population remaining with the set of 6 lowest electronic states.

**Table 2:** Optimal Parameters of the Pulses That Maximize χ_1_ and χ_2_ Using the Linear-Search Approach (LS) and the Gradient-Based Optimization (OC)

	$\chi_{1}^{\left(\mathrm{LS}\right)}$	$\chi_{1}^{\left(\mathrm{OC}\right)}$	$\chi_{2}^{\left(\mathrm{LS}\right)}$	$\chi_{2}^{\left(\mathrm{OC}\right)}$
*E*_1_ (a.u.)	0.0365	0.0369	0.0580	0.0728
ω_1_ (a.u.)	0.1486	0.1947	0.1486	0.1320
σ_1_ (fs)	20	19.35	20	17.95
τ (fs)	4.88	4.72	6.25	5.76
*E*_1_ (a.u.)	0.0089	0.0106	0.0106	0.01218
ω_2_ (a.u.)	0.3428	0.3433	0.3428	0.3338
σ_1_ (fs)	20	19.35	20	17.10

**Figure 8:**
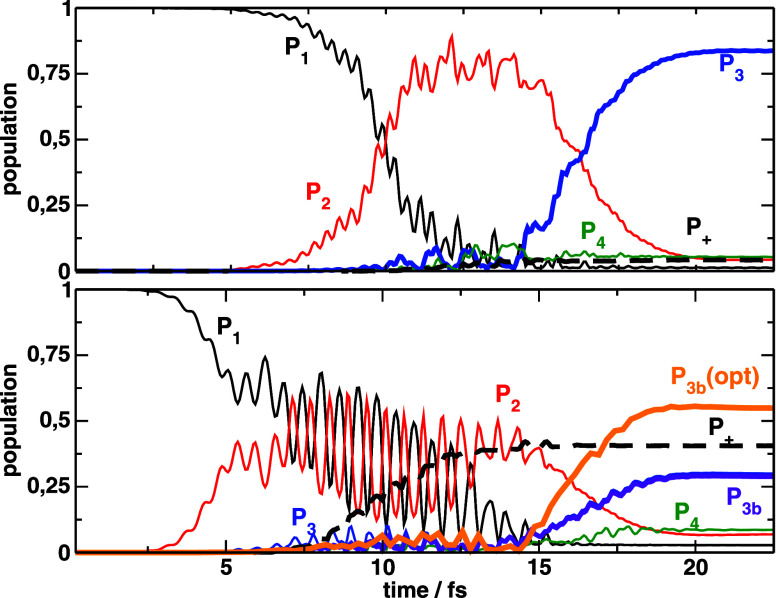
Population dynamics driven by the optimal pulses obtained from a gradient based optimal control algorithm, starting from the wave functions $\psi_{j}^{\left(1\right)}$ , that maximize the final electronic population χ_1_ (top) or only the bounded population χ_2_ (bottom). *P*_3*b*_ refers to the vibrational (bounded) population in the *V*_3_ potential. Maximizing χ_2_ requires more intense pulses, that lead to substantial ionization or population of higher excited electronic states. For reference, we show the bounded population reached using the Hamiltonian model with 6 electronic states (*P*_3*b*_(opt)), from which the optimal pulses and wave functions were found.

The dynamics shown in Figure 8, calculated using the full Hamiltonian, shows again similar dynamics to those depicted previously, with optimized initial wave functions that exhibit the same kind of nodal patterns, mostly in the coordinate representation for $\psi_{1}^{\left(1\right)}$ , and in the momentum representation for $\psi_{2}^{\left(1\right)}$ (Figure 9). This suggests that the underlying physical mechanism that relates the Rabi oscillations to certain nodal patterns can be more general. Further studies will be needed to interpret the results with a proper physical model.

## Conclusions

Combining laser optimization with wave function optimization in an iterative procedure, we have shown that one can obtain high yields of selective electronic excitation after very few iterations.

Targeting population transfer to the second excited state of the hydrogen molecular cation, B ^2^Σ_*g*_, is a very demanding problem that requires controlling population passage through the dissociative electronic state, A ^2^Σ_*u*_, which severely restricts the time duration of the process. In addition, it involves outcompeting multiphoton absorption, favored by large transition dipoles especially at large internuclear distances, dissociation, favored by absorption windows at the highly energetic slope of the potential curve and ionization. However, even when the parameters of the laser pulses were optimized for an initial Gaussian wave packet, we found viable initial wave functions constructed from vibrational states of the ground potential, that could be used to clearly enhance the yields of the desired processes. This shows the potential use of the geometrical optimization to avoid local traps in the landscape of optimal control inducing fast convergence at a lower computational cost.

**Figure 9:**
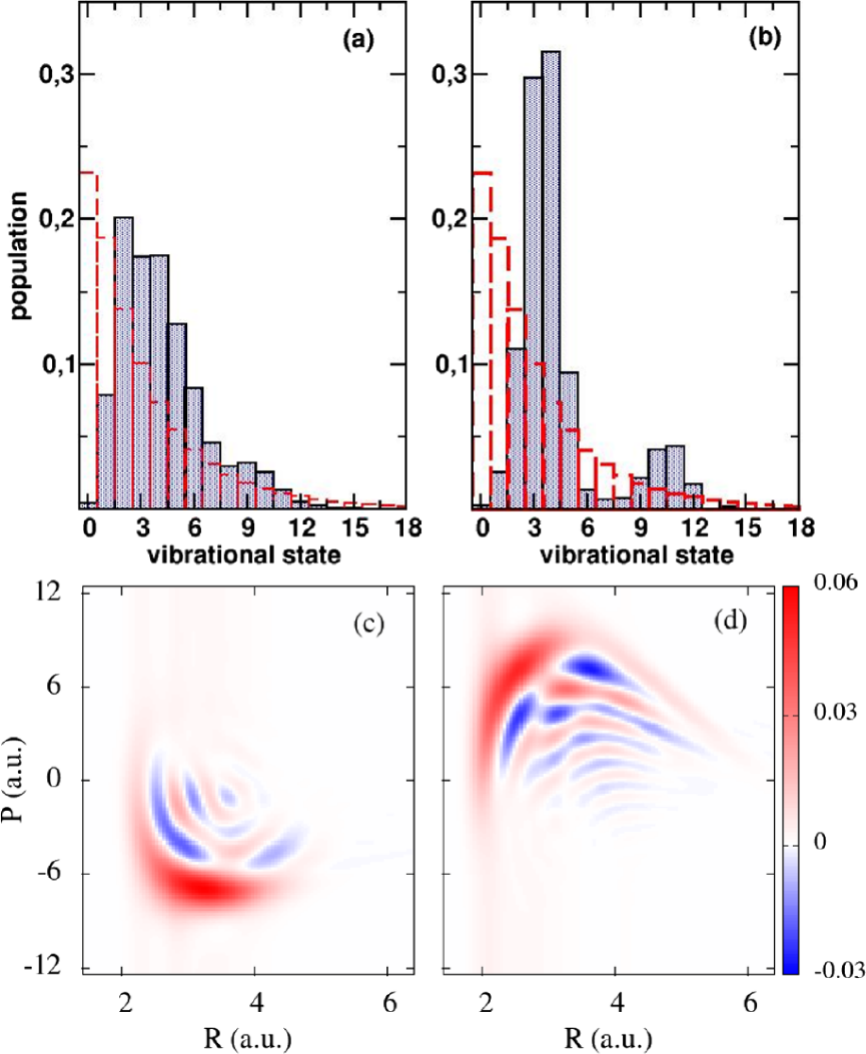
Optimized initial wave functions in the quantum number representations that maximize the final electronic population χ_1_ (a) or only the bounded population χ_2_ (b), obtained using the gradient-based optimal control algorithm. In red-dashed line we show the populations of the initial Gaussian wave packet. The Wigner transforms of the wave functions are shown in panels (c) and (d) for χ_1_ and χ_2_, respectively.

In addition to the possible numerical advantage in using the geometrical optimization, it helps at analyzing which physical resources are more important in the control (e.g., the vibrational coherences) and contributes to finding novel dynamics. In our example, the optimal initial wave functions vary completely depending on whether we maximize all the electronic population or only the vibrational (bound) population of the target electronic state. The observed nodal patterns in either the spatial or momentum domains, hint to novel mechanisms of population transfer that are being used to optimize the dynamics, which will be explored in future studies.

Finally, we would like to comment on some possible practical implementations of our scheme in the laboratory. In the simpler scenarios of the geometrical optimization, one can conceive using a sequence of an IR pulse followed by the vis–UV pulse.^23–28^ However, most practical implementations to prepare the optimized initial wave functions will require using pulse shaping, applying an adaptive feedback genetic algorithm.^10,68–70^ Because the vis–UV pulses that we need are simple, our scheme would imply reverting the central role of the optimization: from the vis–UV to the IR pulse. While currently this change may not be the most convenient for the experiment, because most acousto-optic modulators operate in the near IR^71^ some new techniques have been reported that can be used for pulses with wavelengths up to the far IR, which can excite most typical vibrations in molecules.^72^ In addition, a superposition of vibrational states can also be prepared by stimulated Raman using two ultrashort pulses.^29–74^ In this case, controlling the dynamics would require the use of four pulses, following a pump-dump-pump–pump process, where the second pump could be identical to the first one. The first excursion in the excited dissociative A electronic state could be used to displace the wave packet from the Franck–Condon region, creating the initial superposition, which could be further optimized using pulse shapers and genetic algorithms in the usual manner. Then the second excursion would proceed through the pump–pump process to the target B electronic state.

## Supplementary Material



## Notes

Tables with the complex coefficients of the optimized initial states as superpositions of the vibrational eigenstates of the ground potential, obtained for the linear search of the pulse parameters (Table S1) or the gradient-based optimal control optimization of the parameters (PDF)
