# Supplementary material for: Combining Optical Control and Geometrical Optimization for Efficient Control of Competing Molecular Photoinduced Processes Far from the Ground State
Source: J Chem Theory Comput. 2025 Jun 20;21(13):6315–24. doi: 10.1021/acs.jctc.5c00609 (PMC12243093; doi:10.1021/acs.jctc.5c00609)
Supplement: Supplementary file 1 [file ct5c00609_si_001.pdf]

SUPPORTING INFORMATION

Combining optical control and geometrical optimization for efficient control of competing molecular photo-induced processes far from the ground state

David Veintemillas,[†] Bo Y. Chang,[‡,†], Ignacio R. Sola[†,*]

†Departamento de Química Física, Universidad Complutense de Madrid, 28040 Madrid (Spain)
‡School of Chemistry, Seoul National University, 08826 Seoul, Republic of Korea
*Email: isolarei@ucm.es

Table 1 SI. Coefficients of the optimized initial state that maximizes the yield χ_j,(j = 1 for the total electronic population in V_3; j = 2 for the bound population) in terms of all the vibrational states of the ground electronic state, obtained after the second iteration of algorithm, $\psi_j^{(2)} = \sum_v^{N_1} c_v \phi_v$, where N_1 = 24. The pulse amplitudes and the time delay between both pump pulses were optimized as in typical laser experiments, performing a sequential line search over the parameters.

	$\psi_1^{(2)}$		$\psi_2^{(2)}$	
v	$Re[c_v]$	$Im[c_v]$	$Re[c_v]$	$Im[c_v]$
0	-0.009	-0.010	-0.003	-0.065
1	-0.063	0.053	0.159	0.175
2	-0.109	-0.080	0.521	0.095
3	-0.129	0.035	-0.442	0.507
4	-0.212	0.065	-0.242	-0.369
5	0.044	-0.054	0.075	-0.034
6	0.349	0.241	-0.034	-0.030
7	0.189	0.506	0.009	-0.009
8	-0.125	0.274	-0.004	0.004
9	-0.244	-0.064	-0.005	-0.012
10	-0.280	-0.173	0.008	0.004
11	0.283	0.086	0.013	-0.014
12	0.212	-0.071	-0.002	-0.005
13	0.038	-0.142	0.000	-0.004
14	-0.068	-0.039	0.003	-0.005
15	-0.013	0.026	-0.005	-0.006
16	0.001	-0.002	-0.008	0.005
17	0.000	0.012	0.007	0.006
18	-0.009	0.015	-0.001	0.007
19	-0.015	0.005	-0.004	0004
20	-0.008	0.001	-0.004	0.001
21	0.005	0.000	0.002	-0.000
22	-0.003	0.000	-0.001	0.000
23	0.003	0.000	0.001	0.000

Table 2 SI. Coefficients of the optimized initial state that maximizes the yield χ_j,(j = 1 for the total electronic population in V_3; j = 2 for the bound population) in terms of all the vibrational states of the ground electronic state, $\psi_j^{(1)} = \sum_v^{N_1} c_v \phi_v$, where N_1 = 24. All pulse parameters (amplitudes, frequencies, durations, as well as the time delay between both pump pulses) were optimized using a gradient-based optimal control algorithm.

	$\psi_1^{(1)}$		$\psi_2^{(1)}$	
v	$Re[c_v]$	$Im[c_v]$	$Re[c_v]$	$Im[c_v]$
0	-0.063	-0.015	-0.028	0.046
1	0.203	0.192	0.111	-0.116
2	0.157	0.420	0.161	-0.290
3	0.298	-0.292	0.047	0.544
4	-0.330	-0.255	-0.467	-0.311
5	-0.216	0.283	0.285	-0.112
6	0.221	0.185	-0.086	0.075
7	0.143	-0.159	0.005	-0.084
8	-0.148	-0.086	0.082	0.022
9	-0.087	0.157	-0.060	0.134
10	0.125	0.099	-0.165	-0.115
11	-0.079	0.083	-0.118	0.170
12	0.059	0.042	-0.124	-0.048
13	0.011	-0.047	-0.013	-0.016
14	-0.033	0.010	-0.043	0.012
15	0.021	0.015	-0.006	-0.002
16	-0.002	-0.018	-0.000	0.022
17	-0.007	0.011	0.008	-0.008
18	-0.007	0.004	0.001	0.000
19	-0.005	0.001	-0.003	0.000
20	-0.004	0.000	-0.003	0.000
21	0.002	-0.000	0.002	0.000
22	-0.001	0.000	-0.001	-0.000
23	0.001	0.000	0.000	0.000
